# Quadricuspid aortic valve by using intraoperative transesophageal echocardiography

**DOI:** 10.1186/1476-7120-8-36

**Published:** 2010-09-02

**Authors:** Zhenghua Xiao, Wei Meng, Eryong Zhang

**Affiliations:** 1 Department of Thoracic and Cardiovascular Surgery, West China Hospital, Sichuan University, ChengDu, China

## Abstract

Quadricuspid aortic valve is a rare congenital malformation of the aortic valve. Its diagnosis is often missed even with the use of transthoracic echocardiogram. Many of these patients progress to aortic incompetence later in life, hence requiring surgical intervention.

In the case described in this report, a 61-year-old woman is presented with the features of congestive heart failure. The preoperative transthoracic echocardiogram disclosed a moderate to severe aortic valve insufficiency but failed to reveal the quadricuspid aortic value anomaly. This case underscores the important role of three-dimensional transesophageal echocardiography for the diagnosis of quadricuspid aortic valve.

## Background

Quadricuspid aortic valve (AV) is a very rare congenital anomaly with an incidence of 0.008% at autopsy and 1% in patients presented for AV surgery [1]. In addition, quadricuspid AV can be associated with other congenital cardiac deformities. Hence, early recognition and follow-up is critical in these patients. A case is presented where three-dimensional (3-D) transesophageal echocardiography was used for the diagnosis and identification of the quadricuspid AV.

## Case presentation

A 61-year-old woman was presented to the hospital with shortness of breath, dizziness, and palpitation. Her exercise tolerance was unremarkable until the past 3 - 4 months when she noted an increase in dyspnea and palpitation on mild exertion.

Pertinent exam revealed a water hammer pulse with blood pressure of 110/50 mmHg. Under cardiac auscultation, an early diastolic murmur on the second right intercostal space and a hyperdynamic apical impulse was heard.

**Figure 1:**
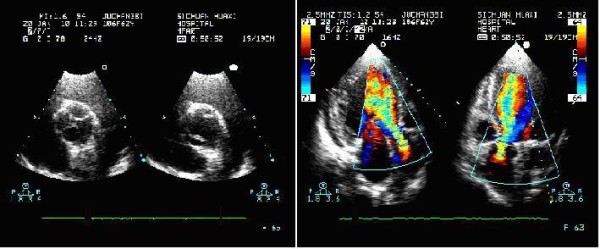
**The preoperative transthoracic echocardiogram (TTE) disclosed a moderate to severe aortic valve insufficiency but failed to reveal the quadricuspid aortic valve anomaly clearly**.

Transthoracic echocardiography (TTE) demonstrated severe aortic insufficiency (AI) with a dilated left ventricular (LV) and a normal function (EF = 55%). Moreover, a suspected quadricuspid AV was seen but there was no confirmation due to bad image quality in this overweight patient. No other pathology was noted (Fig. 1).

In order to rule out other potential congenital deformation and coronary artery and big vessel disease, a CT angiogram was done and showed a severe central AI. The ascending aorta has a mild aneurismal dilatation.

**Figure 2:**
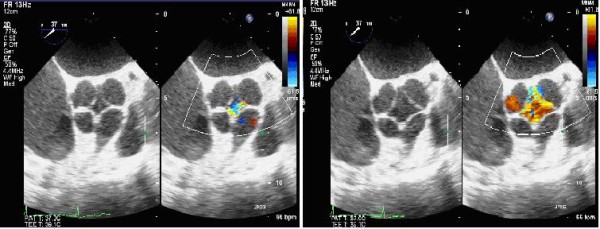
**Transesophageal echocardiogram (TEE) short axis views in the vicinity of the aortic valve plane showed a quadricuspid aortic valve**. The four cusps demonstrated a mild degree of calcification but a well-preserved motion. There was a central jet on the color Doppler examination, which is consistent with the severe aortic insufficiency (AI).

The patient was then scheduled for AV replacement surgery. During the operation, intraoperative transesophageal echocardiogram showed a clear quadricuspid AV consisting of four cusps of equal size. The quadricuspid aortic valve (QAV) was identified by its characteristic "X" configuration during systole (different from the "Y" of the normal tricuspid aortic valve) (Fig. 2).

**Figure 3:**
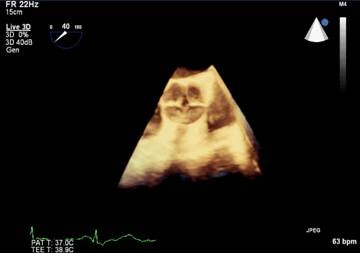
**3-D transesophageal echocardiogram**. The aortic valve opened and closed. It consiste of four cusps of equal size. This was clearer compared with those using 2-D transesophageal echocardiogram.

Meanwhile, miscoaptation of these four aortic leaflets with moderate to severe AR was also confirmed. Furthermore, 3-D echocardiogram revealed degenerated change in the tip of the four aortic leaflets that caused the AR. (Fig 3)

The cusps showed a mild degree of calcification but a well-preserved motion. Furthermore, transesophageal echocardiogram examination showed a normal LV function, moderate LV hypertrophy, and mild aneurismal dilation of the ascending aorta.

The patient underwent a minimally invasive quadricuspid AV replacement with a Medtronic Hancock II porcine valves and ascending aorta forming via a midline sternotomy.

## Discussion

Quadricuspid AV is a very rare congenital anomaly with an incidence of 0.008% at autopsy and 1% in patients presented for AV surgery [1]. The knowledge of the existence of QAV dates back to 1862 with its first description given by Balington [2]. Since then, there have been more than 190 cases reported in literature with echocardiography being the leading mode of detection, followed by surgery, autopsy, and aortography, with detection rates of 51.1%, 22.6%, 15.6%, and 6.5%, respectively [3]. Hurwitz and Roberts classified quadricuspid valves into seven subtypes based on cusp size and degree of cusp equality [4]. The competency of a quadricuspid valve has been shown as related to aortic cusp morphology and symmetry.

The mechanism of this congenital malformation is not fully known. One of the leading hypotheses is an abnormal septation of embryological truncus arteriosus. Normally, after the septation of the arterial trunk, three mesenchymal swellings develop into semilunar leaflets of the aortic and pulmonary trunk. However, in quadricuspid aortic valve, the fourth cusp arises during the early stage of truncal septation resulting to either a different number of primordial aortic leaflets or an abnormal cusp proliferation [5].

QAV may be isolated or associated with other congenital cardiac abnormalities, including coronary ostium displacement or obstruction, altered coronary artery anatomy, ventricular septal defect, patent ductus arteriosus, subaortic fibromuscular stenosis, and malformations of the mitral valve [6]. Nowadays, TTE plays an important role in diagnosing this anomaly [7,8]. Meanwhile, clinical awareness should be raised for this rare congenital heart disease. Other than careful echocardiogram exams, CT angiogram (or even invasive coronary angiogram) is also necessary to preclude potential associated abnormalities. For this 61-year-old female patient, the CT shows no other congenital malformation or coronary artery disease other than QAV.

TEE provided a clearer visualization of the heart internal structure as compared with routine TTE. However, in some instances, TTE may be suboptimal to recognize this malformation and the origin of the coronary vessel due to bad acusted window especially for overweight patients. As shown in this patient, TTE only showed the severe AR but did not confirm the diagnosis of QAV.

In contrast, the intraoperative TEE showed more sensitivity and specificity in diagnosing the QAV compared with TTE [1,3]. TEE, especially 3-D TEE, provided a clear vision for QAV and its pathological change.,

Meanwhile, the key information provided by TEE, especially 3 D TEE, is not only for diagnosis purposes, but also as a guide for surgical procedures. As shown in this patient, miscoaptation is present in both four leaflets of the aortic valve. Instead of valve repair, replacement with bioprothesis may be a better choice for this patient.

## Conclusion

The advent of echocardiography has increased greatly the frequency of detection. Sometimes, it may not be possible to visualize the aortic leaflets adequately with the transthoracic echo, but the intraoperative TEE produces images clearer than TTE. As it is in our case, TEE cannot only produce images of the QAVs clearly, but also uses the 3-D image to reconstruct the QAVs opening and closing conditions. Thus, it plays an important role in reaching the appropriate diagnosis and in guiding further surgical procedure.

## Abbreviations

TTE: transthoracic echocardiography; LV: left ventricle; AI: aortic insufficiency; QAV: quadricuspid aortic valve; CT: computed tomography; AV: aortic valve; TEE: transesophageal echocardiogram.

## Competing interests

The authors declare that they have no competing interests.

## Consent

A written informed consent was obtained from the patient's legal representative for the publication of this case report and any accompanying images.

## Authors' contributions

ZX conceived the case report, performed bedside echocardiographic examinations, reviewed literature, and wrote the manuscript. ZX, WM, and EZ have been involved in drafting the manuscript. WM and EZ contributed critical revisions of the manuscript. EZ is the head of the Department of Thoracic and Cardiovascular Surgery, West China Hospital, Sichuan University, and made significant revisions in the manuscript. All authors read and approved the final manuscript.
